# Stokes flow singularities in a two-dimensional channel: a novel transform approach with application to microswimming

**DOI:** 10.1098/rspa.2013.0198

**Published:** 2013-09-08

**Authors:** Darren G. Crowdy, Anthony M. J. Davis

**Affiliations:** 1 Department of Mathematics, Imperial College London, 180 Queen's Gate, London, SW7 2AZ, UK; 2 Mechanical and Aerospace Engineering, University of California, San Diego, La Jolla, CA 92093-0411, USA

**Keywords:** Stokes flow, transform method, spectral analysis

## Abstract

A transform method for determining the flow generated by the singularities of Stokes flow in a two-dimensional channel is presented. The analysis is based on a general approach to biharmonic boundary value problems in a simply connected polygon formulated by Crowdy & Fokas in this journal. The method differs from a traditional Fourier transform approach in entailing a simultaneous spectral analysis in the independent variables both along and across the channel. As an example application, we find the evolution equations for a circular treadmilling microswimmer in the channel correct to third order in the swimmer radius. Significantly, the new transform method is extendible to the analysis of Stokes flows in more complicated polygonal microchannel geometries.

## 1. Introduction

The study of low Reynolds number flows in confined geometries bounded by no-slip walls is of perennial interest in fluid dynamics [1]. It has received a resurgence of attention in recent years owing to new applications in emerging technologies in micro- and nanofluidics, biological fluid dynamics and colloid science where the small scales render the role of inertia negligible. The relevant boundary value problems are linear but finding solutions even for the fundamental singularities of Stokes flows in just two dimensions can be challenging if the geometries are complicated. The monograph by Pozrikidis [1] documents solutions for the fundamental Stokes singularities in various geometries and surveys the variety of mathematical techniques available for their analysis.

This paper focuses on Stokes flow in the relatively simple geometry of a two-dimensional channel, but it offers a new mathematical perspective. The geometry is an important one in applications. Davis [2] has presented the solutions to various problems involving distributions of point forces, or stokeslets, in a two-dimensional channel with a view to understanding the blocking properties of periodic arrays of wall-attached barriers. His analysis makes use of Fourier transforms with respect to the variable along the channel length. Liron & Mochon [3] have found the Stokes flow field due to a three-dimensional force singularity at an arbitrary location and arbitrary orientation between two parallel plates. Section 3.5 of the monograph by Pozrikidis [1] describes a standard Fourier transform analysis of a two-dimensional point force in a channel.

Although it is not the principal focus of this article, the present study was motivated by problems of computing the swimming dynamics of low Reynolds number organisms in microchannels. In a recent paper, the authors [4] have given a general prescription for computing the dynamical system governing the motion of a model two-dimensional circular treadmilling swimmer in an arbitrary domain confined by no-slip walls. By an asymptotic expansion, the governing ordinary differential equations are determined correct to third order in the swimmer radius: for a given fluid region, rather than solving a boundary value problem for the full finite-area swimmer, one solves boundary value problems for certain point singularities of Stokes flow—namely, a torque-free stresslet and an irrotational (source) quadrupole—in that domain. Even though this is usually simpler than solving the full boundary value problem for the finite-area swimmer, it remains mathematically challenging for most geometries and this challenge has led to the results herein.

Our principal aim here is to present a transform approach to finding analytical solutions for those singular Stokes flows in the channel geometry. We adapt a new general transform approach to biharmonic boundary value problems in a simply connected polygon formulated by Crowdy & Fokas [5], who originally showcased their method for canonical semi-strip problems in plane elastostatics. The method differs from the usual Fourier transform approach where transforms are taken only with respect to the independent variable along the channel length [1,2]; instead, a *simultaneous* spectral analysis with respect to the two independent variables both along and across the channel is performed. It offers various mathematical advantages, not least of which is the fact that the approach can be generalized to *any* simply connected polygonal geometry. This work appears to be the first to apply the general method of [5] to problems of slow viscous Stokes flows, where biharmonic boundary value problems also arise. It showcases the method in one of the simplest non-trivial situations (the channel geometry) and therefore constitutes a valuable case study. The simplicity of the geometry also allows an instructive comparison with traditional Fourier transform methods detailed in an appendix. As an illustrative example, in §5, we apply the method to our motivating problem of treadmilling microswimmers in a channel.

## 2. Stokes flows in two dimensions

Consider a region of incompressible fluid of viscosity *μ* evolving according to the Stokes equations. It is well known that a solution of the Stokes equations for the incompressible velocity field (*u*,*v*) can be written in terms of a streamfunction *ψ*(*x*,*y*) with
2.1
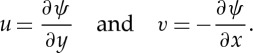
The streamfunction then satisfies the biharmonic equation
2.2
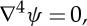
where ∇^2^ is the two-dimensional Laplacian. Crowdy & Fokas [5] present their approach to biharmonic boundary value problems in a polygon in terms of *ψ* and its various partial derivatives, but there is an alternative equivalent presentation of the same method in terms of the so-called *Goursat functions* [6]. With *z*=*x*+*iy* the Goursat functions, here denoted by *f*(*z*) and *g*(*z*), are analytic functions of *z* in the fluid region with the property that the solution of (2.2) is represented in the form
2.3
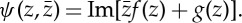
The Goursat functions are familiar to fluid dynamicists using complex variable methods for Stokes flows [6] so we adopt this formulation and seek integral representations of the required physical quantities *u*,*v*, fluid pressure *p* and fluid vorticity *ω* by making use of the following relations of the latter quantities to the Goursat functions [6]:
2.4
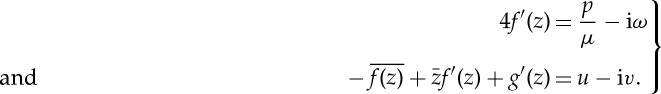


## 3. Point stresslet in a channel

One important singularity of Stokes flow is the *stresslet* [1]. Consider a channel occupied by fluid at zero Reynolds number in the region 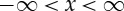, 0<*y*<*h*. A torque-free stresslet of strength 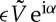 at a point *z*_0_ requires that *f*(*z*) and *g*′(*z*) have the local expansions [7]
3.1
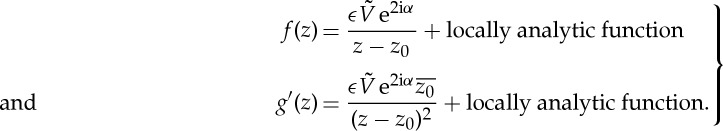
Our objective is to find the flow induced in the channel by such a singularity. The velocity as 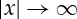 is taken to vanish. We focus on this stresslet for two reasons: first, it is relevant to the study of swimming organisms considered later; second, we have been unable to find the solution reported elsewhere (although it can in principle be derived as an appropriate parametric derivative, with respect to the singularity position, of the solution for a point stokeslet in a channel given by Davis [2]). It should be pointed out that the quantity 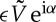 is just a complex constant but its designation has been chosen so as to be consistent with our later application of the results to a model treadmilling swimmer.

It is convenient to introduce the meromorphic functions
3.2
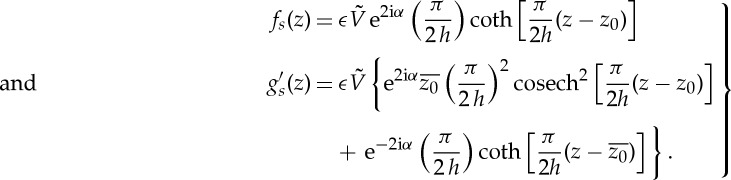
The functions *f*_*s*_(*z*) and *g*_*s*_′(*z*) have precisely the required local form (3.1) at *z*_0_ and no other singularities in the channel. Both functions are periodic with period 2 h*i*, i.e.
3.3
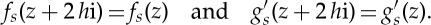
The derivative *f*_*s*_′(*z*) is also 2 h*i*-periodic and this will be useful later.

We now pose the ansatz
3.4
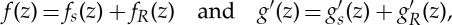
where *f*_*R*_(*z*),*g*_*R*_′(*z*) are analytic in the channel and vanish as 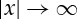; note that the second term in the definition of *g*_*s*_′(*z*) has been chosen to ensure that
3.5
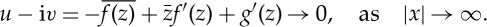
It should be emphasized that it is not necessary to introduce the hyperbolic functions (3.2) and one can proceed instead by defining *f*_*s*_(*z*) and *g*_*s*_′(*z*) to have the simple rational function forms in (3.1). However, we have found that the periodicity properties (3.3) greatly simplify the algebraic manipulations to follow.

The no-slip conditions on the two channel walls are
3.6
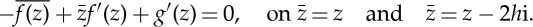
On substitution of the ansatz (3.4), we find that, on 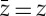,
3.7
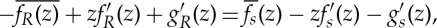
and, on 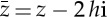,
3.8
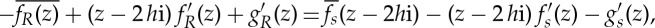
where known functions appear on the right-hand sides of (3.7) and (3.8). The Schwarz conjugate of an analytic function *h*(*z*) is defined by
3.9
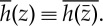
Following the general formulation in [5] the two functions *f*_*R*_(*z*) and *g*_*R*_′(*z*), which are analytic in the channel and decaying in the far-field, will be represented by
3.10
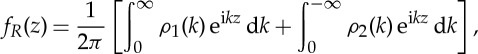
where *ρ*_1_(*k*) and *ρ*_2_(*k*) are defined by
3.11
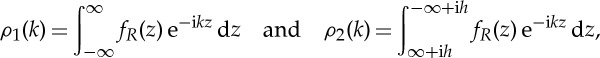
and, similarly,
3.12
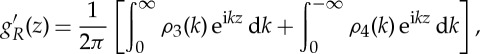
where *ρ*_3_(*k*) and *ρ*_4_(*k*) are defined by
3.13
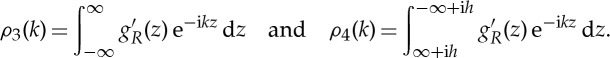
The set {*ρ*_*n*_(*k*)|*n*=1,2,3,4} is called the set of *spectral functions* [5,8], and there are relations between these spectral functions referred to as *global relations*. In this case, there are two global relations given by
3.14
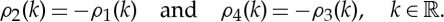
In this case, these relations can be viewed as a consequence of Cauchy's theorem: for example, the condition *ρ*_2_(*k*)=−*ρ*_1_(*k*) is equivalent to the statement that the integral of *f*_*R*_(*z*) e^−i*kz*^ around the channel is zero because, for 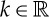, the latter function is analytic inside the channel. On use of these relations (3.10) can be written as
3.15
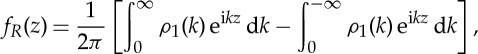
or, on making the substitution *k*↦−*k* in the second integral,
3.16
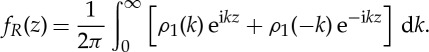
Similarly, we can write
3.17
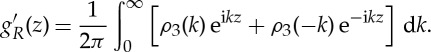
It is worth emphasizing that it is this spectral representation of the analytic functions *f*_*R*_(*z*) and *g*_*R*_′(*z*) that can be extended to arbitrary simply connected polygons [5,8,9].

### (a) Preliminary observations

The representations of *f*_*R*_(*z*) and *g*_*R*_′(*z*) above involve only real values of *k*. Hence, for real *k*, complex conjugation implies
3.18
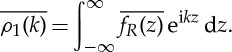
If we let *k*↦−*k* we find
3.19
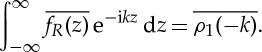
The integral on the left-hand side will appear in the transform of the boundary conditions on the wall at *y*=0.

Similarly, on writing *x*=*z*−*ih* in the integral expression for *ρ*_2_(*k*), we can write
3.20
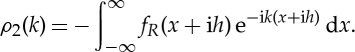
On taking a complex conjugate for real *k*,
3.21
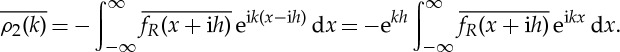
Now making the substitution *x*=*z*−*ih* in the last integral produces
3.22
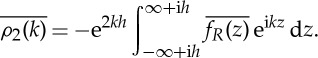
Hence, mapping *k*↦−*k*, it follows that
3.23
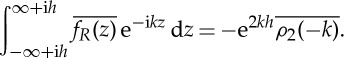
Again, the integral on the left-hand side will appear in the transform of the boundary conditions on the wall at *y*=*h*.

### (b) Relations between spectral functions

The boundary conditions on the channel walls allow us to deduce more information about the spectral functions. To proceed, we multiply the boundary condition (3.7) by e^−i*kz*^ and integrate along the real *z*-axis:
3.24
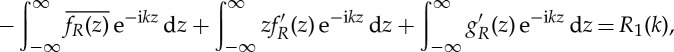
where
3.25
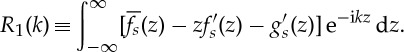
The function *R*_1_(*k*) is known. On use of the result, available from an integration by parts,
3.26
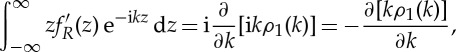
together with (3.19), we find
3.27
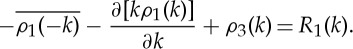
Similarly, if we multiply the second boundary condition (3.8) by e^−i*kz*^ and integrate over the upper boundary, we find
3.28
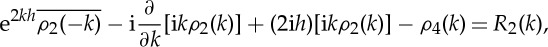
where
3.29
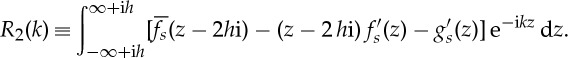
Equations (3.27) and (3.28) can be analysed, together with the global relations (3.14), to determine the spectral functions.

Indeed, on use of (3.14) equation (3.28) becomes
3.30
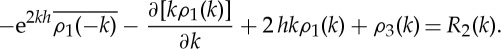
On subtraction of (3.27) from (3.30), we find
3.31
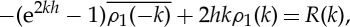
where
3.32
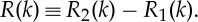
After taking a complex conjugate and letting *k*↦−*k*, we find
3.33
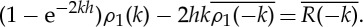
Elimination of 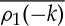 from (3.31) and (3.33) produces
3.34
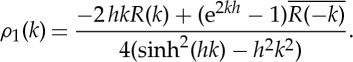
The function *R*(*k*) can be computed explicitly using residue calculus to give
3.35
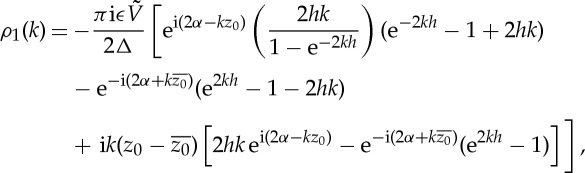
where
3.36
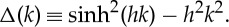
Given *ρ*_1_(*k*), the spectral function *ρ*_3_(*k*) follows from (3.27) together with
3.37
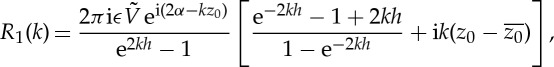
which also follows from a residue calculation. All physical quantities are now available from the representations, dependent only on *f*_*s*_(*z*),*g*_*s*_′(*z*) and the associated spectral functions *ρ*_1_(*k*) and *R*_1_(*k*), given by
3.38
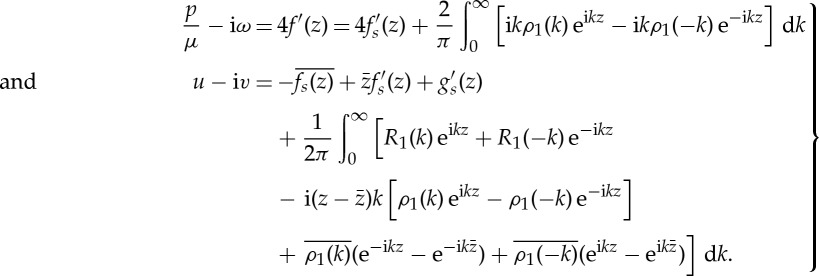
This is the required solution. Significantly, these integrals are readily computed numerically owing to the exponential decay of all integrands as 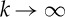. As *k*→0,
3.39
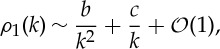
where
3.40
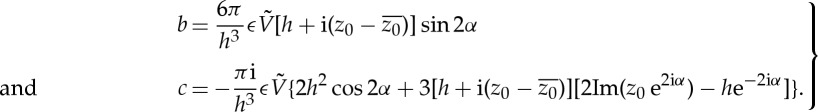
Note that *b* is real-valued and *c* becomes purely imaginary if *y*_0_=*h*/2 or 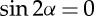. Expressions (3.40) can be used to verify that all integrands in (3.38) are regular at *k*=0. Formula (3.39) is also useful in facilitating explicit computation of the limiting behaviour of the integrands in any numerical evaluation procedure. Finally, it should be pointed out that although the integrals defining all physical quantities in (3.38) are regular at *k*=0 the integrand in the expression (3.16) for *f*_*R*_(*z*) is singular at *k*=0 but this is rendered immaterial by a freedom to redefine *f*(*z*)↦*f*(*z*)+*pz*+*q* and 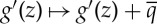 for constants 
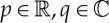
 without affecting any of the physical quantities.

It is instructive, for purposes of comparison, to solve the problem of a point stresslet in a channel using a more traditional approach involving a Fourier transform only with respect to *x*. This separate analysis in appendix A also affords us a numerical check on (3.38).

## 4. Point quadrupole in a channel

It is straightforward to adapt the approach to other singularities. Indeed, the solution for a *stokeslet* singularity [1] in a channel can be derived by simple adaptation of the above approach and compared with the result of Davis [2], who employed standard Fourier transform methods akin to those used in appendix A for the stresslet problem. For use in §6, consider the separate problem in which an irrotational quadrupole is situated at *z*_0_ with the corresponding function *f*(*z*) analytic at *z*_0_ and *g*′(*z*) behaving like
4.1
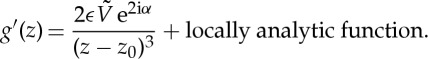
A derivation of the flow generated by this singularity closely follows that of §3: the functional forms in (3.38) are exactly the same, the only difference is that *f*_*s*_(*z*),*g*_*s*_′(*z*) must be adapted thereby producing correspondingly different spectral functions *ρ*_1_(*k*) and *R*_1_(*k*) (it is convenient here to use the same notation as in §3). But it turns out that we can bypass a full analysis by noticing that the functions *f*(*z*) and *g*′(*z*) relevant for the point quadrupole problem can be derived from those of §3 by computing the mixed parametric derivatives
4.2
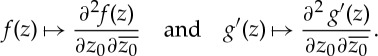
This generation of the flow due to a quadrupole by taking parametric derivatives with respect to the singularity position is equivalent to use of the 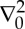 operator by Davis & Crowdy [4] in their analysis of low Reynolds number swimmers in confined domains (the application to be considered in §5). It follows that, for the point quadrupole problem,
4.3
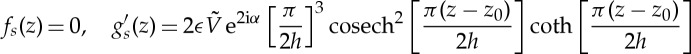
with spectral functions in the integral representations for the corresponding *f*_*R*_(*z*) and *g*_*R*_′(*z*) now given by
4.4
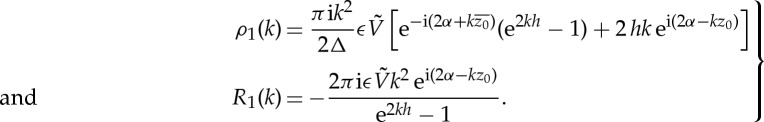


## 5. Application: microswimmers in a channel

One application of the foregoing analysis is to modelling the low Reynolds number swimming of micro-organisms in a channel. In 1963, Lord Rothschild [10] conducted an experiment in which a sample of bull sperm in a very viscous fluid was placed between two glass plates; after time he measured the distribution of the sperm between the two plates and found a tendency for them to aggregate at the channel walls. The experiment was repeated recently by Berke *et al.* [11] and a hydrodynamic explanation of the wall attraction phenomenon proposed. But more general dynamical behaviour of low Reynolds number swimmers near walls has been observed, including steady translation and periodic motions along the walls [12,13]. To rationalize this behaviour using simple mathematical models, Crowdy & Or [7] have proposed the study of a simple circular ‘treadmiller’ comprising a cylindrical circular swimmer with an imposed tangential velocity profile; this profile actuates motion in the spirit of an ‘envelope model’ of surface ciliatory motion [14]. Crowdy & Or [7] showed that, in free space, such a swimmer has the singularity distribution comprising a torque-free stresslet and a superposed irrotational quadrupole; as an approximation, they studied the motion of such a singularity combination near a straight no-slip wall and found good qualitative agreement with numerical [12] and laboratory [15] experiments. Davis & Crowdy [4] have since found the general evolution equations for the same swimmer correct to 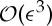, where *ϵ* is the swimmer radius. We now use the latter equations to study the dynamics of such a treadmilling swimmer in a channel.

**Figure 1.**
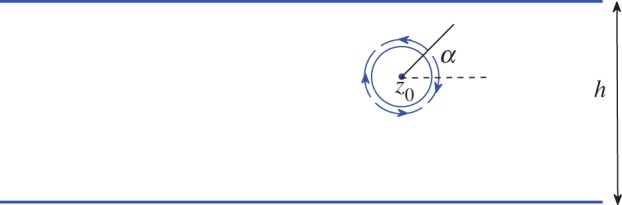
Circular treadmilling swimmer, of the type proposed in [7], centred at *z*_0_(*t*) at orientation *α*(*t*) in a channel of width *h*. The radius of the swimmer is *ϵ*≪1. Its evolution equations, correct to 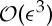, can be determined from the results in [4] using the transform results of §§3 and 4. (Online version in colour.)

The swimmer, which is oriented at some angle *α*(*t*) to the real axis, has an imposed tangential surface velocity of the functional form 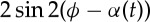, where *ϕ* is an angular coordinate around the swimmer boundary. The interaction of the Stokes flow it generates with any nearby solid walls generally results in dynamical evolution of its centre *z*_0_(*t*). A schematic of such a swimmer located in a channel of width *h* is shown in figure 1.

The evolution equations given in Davis & Crowdy [4] have the following form. Suppose *ψ*_*R*_ denotes the streamfunction of the reflected velocity generated by the wall interaction of the stresslet considered in §3, where
5.1
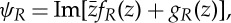
with
5.2
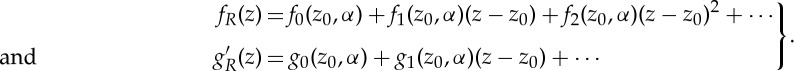
Suppose too that the streamfunction 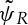 for the reflected velocity generated by the point quadrupole of §4 can similarly be determined in the form
5.3
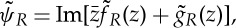
where, near *z*_0_,
5.4
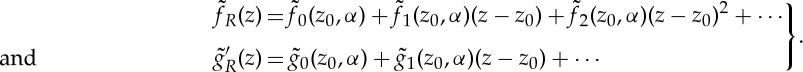
The evolution of the swimmer, correct to 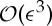, is given [4] by the solution of the system
5.5
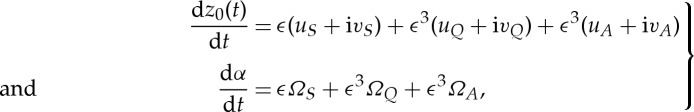
where
5.6
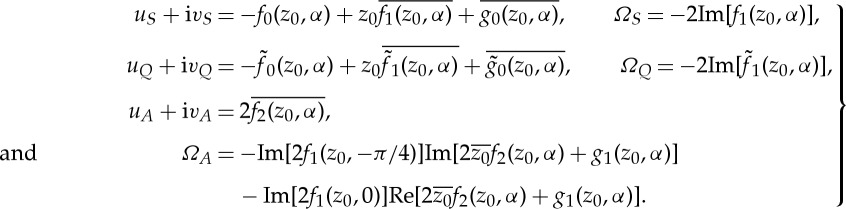
The main point here is that the analysis of §§3 and 4 allow all the quantities (5.6) appearing in the approximate evolution equations (5.5) to be calculated and, thus, the swimmer motion determined.

**Figure 2.**
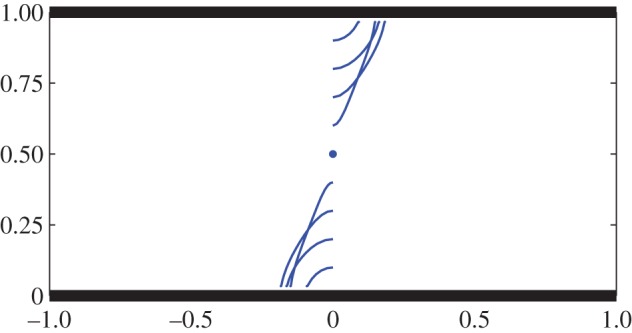
Swimmer attraction to channel walls with *h*=1: trajectories for *ϵ*= 0.03 with initial conditions *α*(0)=*π*/4 for *x*(0)=0,*y*(0)=0.1(0.1)0.9. The swimmer remains stationary when *y*(0)=0.5. The evolution has been computed correct to 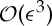 from the asymptotic equations given in [4]. (Online version in colour.)

Figure 2 shows some typical swimmer trajectories for *α*(0)=*π*/4 and *x*(0)=0,*y*(0)=0.1(0.1)0.9 for *h*=1. Consistent with the experimental observations of wall aggregation [10,11], it is found that the swimmer is attracted to the nearest wall of the channel with only swimmers exactly positioned on the channel centre-line remaining stationary. A range of more general initial orientations were explored and all swimmers off the centre-line are similarly found to be attracted to the nearest wall. We found no initial conditions for which the swimmer propagates along the channel. It is important to recall that these evolution equations are approximate and only correct to 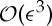; it would be of interest, for purposes of comparison, to compute the full unapproximated evolution equations but this would probably require a numerical treatment using, for example, boundary integral methods [1].

## 6. Discussion

Although our motivating application was the dynamics of low Reynolds number swimming organisms, the principal contribution of this paper is mathematical: a novel transform approach to finding the flows due to basic point singularities of the Stokes equations in a simple two-dimensional channel has been given. Other types of Stokes flow in the channel can be studied using adaptations of the same approach.

The solutions can be derived using alternative approaches, such as traditional Fourier methods, but the method here, based on a general formulation by Crowdy & Fokas [5], is significant in that it can be generalized to finding flows in more complicated simply connected polygonal domains (a similar solution scheme for the Laplace equation in a polygon has been given by Fokas & Kapaev [9]). In those cases, the determination of *f*(*z*) and *g*′(*z*) will follow similar steps: a convenient form of the singular terms *f*_*s*_(*z*) and *g*_*s*_′(*z*) must be chosen based on considerations from the geometry being analysed and the integral representations for the correction terms *f*_*R*_(*z*) and *g*_*R*_′(*z*) will be similar except that every additional side of the polygonal domain will introduce a new spectral function. The set of spectral functions will again satisfy a global relation which must be analysed to determine any unknown spectral functions [5,8]. Here, the global relations are manifested in equations (3.14). In many cases, the analysis of these involves the solution of a Riemann–Hilbert problem for the spectral functions [5,8] but the problem in a channel geometry studied here does not require any such Riemann–Hilbert analysis and the spectral functions can be determined using only algebraic manipulations. Other boundary value problems for which a Riemann–Hilbert analysis can be avoided, and only algebraic manipulations used, have been reported by Fokas [8]. The latter monograph also provides an interesting survey of how the transform method for Stokes flow used here fits into a unified framework for boundary value problems developed in recent years by Fokas and collaborators.

Extensions of our approach to finding Stokes flow solutions, and studying microswimmer dynamics, in more complicated polygonal channel geometries are currently under investigation.

## Appendix A. Standard Fourier transform approach

In this appendix, the problem for a point stresslet in a channel is solved using standard Fourier transform methods. Suppose a stresslet of strength e^2i*α*^ is at *z*_0_=*iY* . The streamfunction *ψ* can be written as

A1

where  is regular in the channel. It is convenient to introduce image singularities in order to satisfy the no normal flow conditions on the walls. Now

A2

and

A3

We therefore write

A4

where

A5

and

A6

Then

A7

Now

A8

by use of the periodic *δ*-function. Similarly,

A9

Hence

A10

The boundary conditions on the channel walls now take the form

A11

Next, we introduce the Fourier sine and cosine transforms defined by

A12

Conditions (A7) imply that

A13

The identity

A14

implies the results

A15

which can be used to take the transforms of (A11)

A16

The Fourier transform of the biharmonic equation satisfied by the streamfunction implies that

A17

Independent solutions of this ordinary differential equation that vanish on the channel walls at *y*=0,*h* are

A18

However, inspection of (A16) shows that, for each function, odd/even values of *n* yield the symmetric/antisymmetric parts with respect to the midplane *y*=*h*/2. Hence we let

A19

where *A*^(*e*)^(*λ*),*A*^(*o*)^(*λ*),*B*^(*e*)^(*λ*) and *B*^(*o*)^(*λ*) are to be determined. Note that both functions remain bounded as *λ*→0. Substitution of (A19) into (A16) then yields

A20

and

A21

Finally, the inverse cosine and sine transforms give

A22

and

A23

The velocity components can be found by differentiation of these expressions. It can be verified numerically that the values obtained are the same as those computed from (3.38).
